# Life-Threatening Bleeding Following a Stable Fracture of the Superior Pubic Ramus: A Case Report

**DOI:** 10.7759/cureus.61520

**Published:** 2024-06-02

**Authors:** Zeinab Al-Rawi, Yasmin Nached, Abdulla Abdelwahab, Baher M Samy

**Affiliations:** 1 Department of Orthopaedics and Trauma, Mohammed Bin Rashid University of Medicine and Health Sciences, Dubai, ARE; 2 Department of Orthopaedics and Trauma, Dubai Health, Rashid Hospital, Dubai, ARE

**Keywords:** conservative treatment, arterial embolization, low-energy pelvic trauma, stable pelvic fracture, corona mortis

## Abstract

Corona mortis, an anatomical variant documented in the literature, presents a noteworthy concern due to its proximity to the superior pubic ramus. Consequently, it remains susceptible to injury, even in stable, benign fractures of the pelvis, typically addressed through conservative management. Stable pelvic fractures are infrequently associated with complications; therefore, diligent monitoring is often overlooked in clinical practice. However, it becomes crucial, particularly in the elderly population given their suboptimal hemostatic capabilities. The standard approach for managing bleeding associated with pelvic fractures involves superselective embolization, a minimally invasive procedure with favorable outcomes. We present a case involving a 61-year-old female who experienced a stable pelvic fracture following low-energy trauma. Despite the ostensibly benign nature of the fracture, the patient exhibited hemodynamic instability attributable to bleeding from the corona mortis, necessitating embolization. The pelvic fracture itself was managed conservatively, leading to the patient's subsequent discharge in a stable condition. Therefore, we advocate for a comprehensive physical examination, serial hemoglobin monitoring, and additional imaging modalities based on the patient's clinical condition.

## Introduction

Corona mortis, translated as "crown of death," is a vascular connection situated posterior to the superior pubic ramus (SPR). This anastomosis occurs between the inferior epigastric artery (IEA), a branch of the external iliac artery (EIA), and the obturator artery (OA), a branch of the internal iliac artery (IIA). Additionally, it may also originate directly from the EIA [1]. This anastomosis can be venous, arterial, or combined [2]. The term "corona mortis" is employed to describe the potential danger at this anatomical site, which may lead to a severe and life-threatening hemorrhage in the event of injury or fracture [3,4].

Stable pelvic fractures are common among elderlies, often managed conservatively with mobilization based on pain tolerance [5]. Complications, particularly those involving arterial injury, are infrequent. Pelvic hemorrhage is typically addressed through preperitoneal packing or superselective embolization, with the latter being the preferred method [6-8]. In this report, we present a unique case involving an elderly female who experienced a nondisplaced stable fracture of her right pubic ramus following a low-energy trauma. This fracture resulted in injury to the corona mortis, causing extraperitoneal hemorrhage and subsequent hypotension.

## Case presentation

A 61-year-old female was brought to the emergency department by her family after a fall from a standing height that resulted in a direct impact on her left hip. This impact caused bilateral hip and lower abdominal pain. The patient reported no loss of consciousness, nausea, or vomiting. Her medical history was significant for hypercholesterolemia, with an otherwise unremarkable health profile.

Upon physical examination, the patient exhibited hemodynamic instability, evident by a drop in blood pressure from 109/70 to 90/57 mmHg, a pulse rate of 108 beats per minute, and a maintained oxygen saturation level of 100%. Chest and cardiac evaluation yielded no findings. Discernible tenderness was observed in the lower abdominal area, and pelvic examination indicated stability with bilateral tenderness, particularly accentuated on the left side. The focused assessment with sonography for trauma (FAST) scan was negative. The hemoglobin level was 11.4 g/dL, and the international normalized ratio was 1.16 at the presentation time.

Given the deterioration of the patient's vital signs, a full polytrauma code was activated, calling the expertise of general surgery, orthopedics, and trauma teams. In a swift response, the patient underwent a transfusion of one unit of un-crossmatched packed red blood cells to address the declining blood pressure expeditiously. Additionally, an interventional radiologist was called and kept on standby to provide timely support as needed.

**Figure 1:**
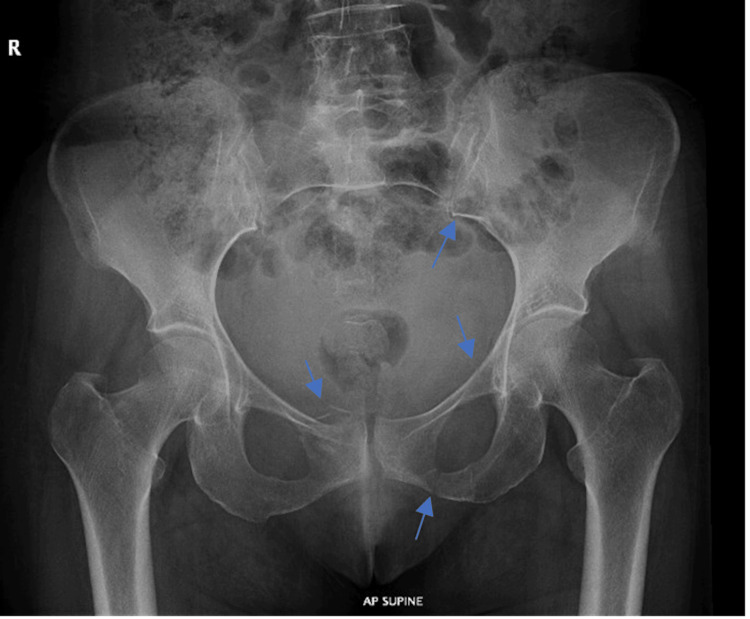
X-ray of the pelvis, showing a fracture of the left sacral ala, right SPR, left SPR, and inferior pubic rami (blue arrows) SPR: superior pubic ramus; AP: anteroposterior

**Figure 2:**
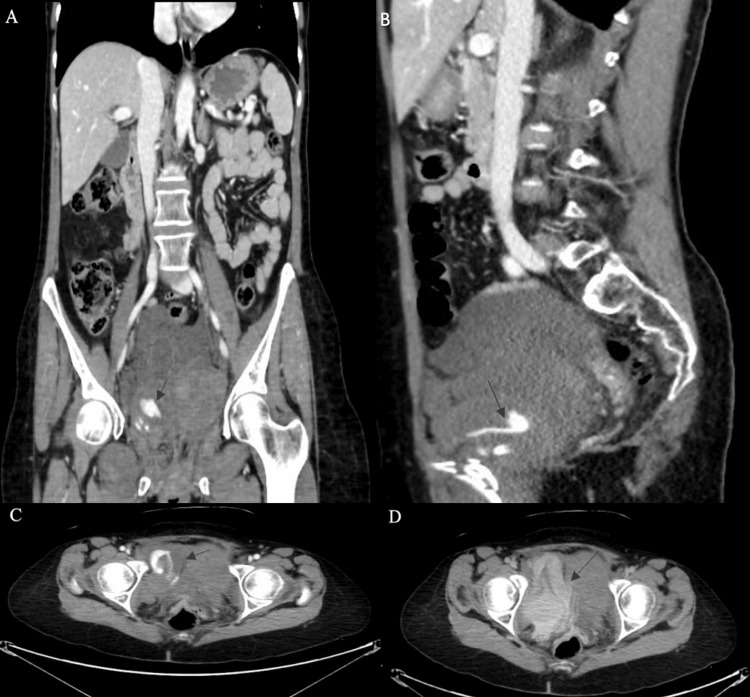
CT of the abdomen and pelvis with contrast showing a substantial pelvic hematoma in the coronal and sagittal views (A,B), while in the transverse sections (C,D), the presence of contrast extravasation within the hematoma unequivocally indicates an ongoing active hemorrhage (arrows) CT: computed tomography

Upon stabilization of the patient, the patient was promptly transferred to the computed tomography (CT) polytrauma room. Subsequently, she underwent a pelvic X-ray (Figure 1) and CT of the abdomen and pelvis with contrast (Figure 2). The CT imaging revealed moderate-to-severe free fluid collection within the pelvis and cul-de-sac, with extravasation of contrast observed in both the early and delayed phases, highly suggesting active arterial bleeding. Additionally, the imaging showed a fracture of the anterior portion of the right SPR and fractures in the left SPR and inferior pubic rami (IPR), along with a concurrent fracture in the left sacral ala. In accordance with the Young-Burgess classification, the patient was diagnosed with a type I lateral compression fracture.

**Figure 3:**
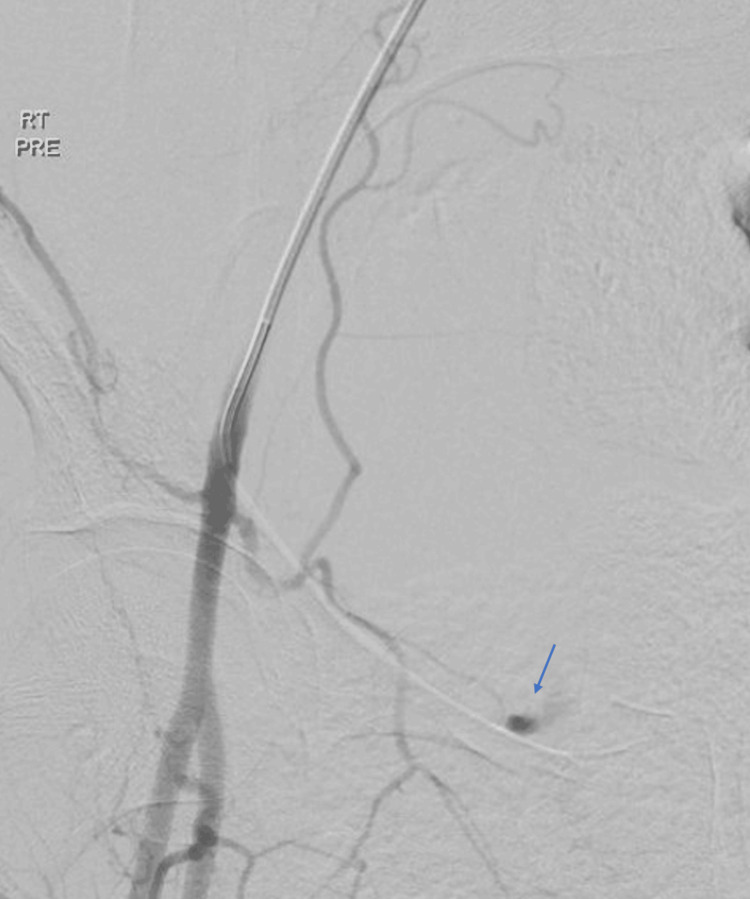
Angiography showing an active extravasation at the right corona mortis (arrow)

**Figure 4:**
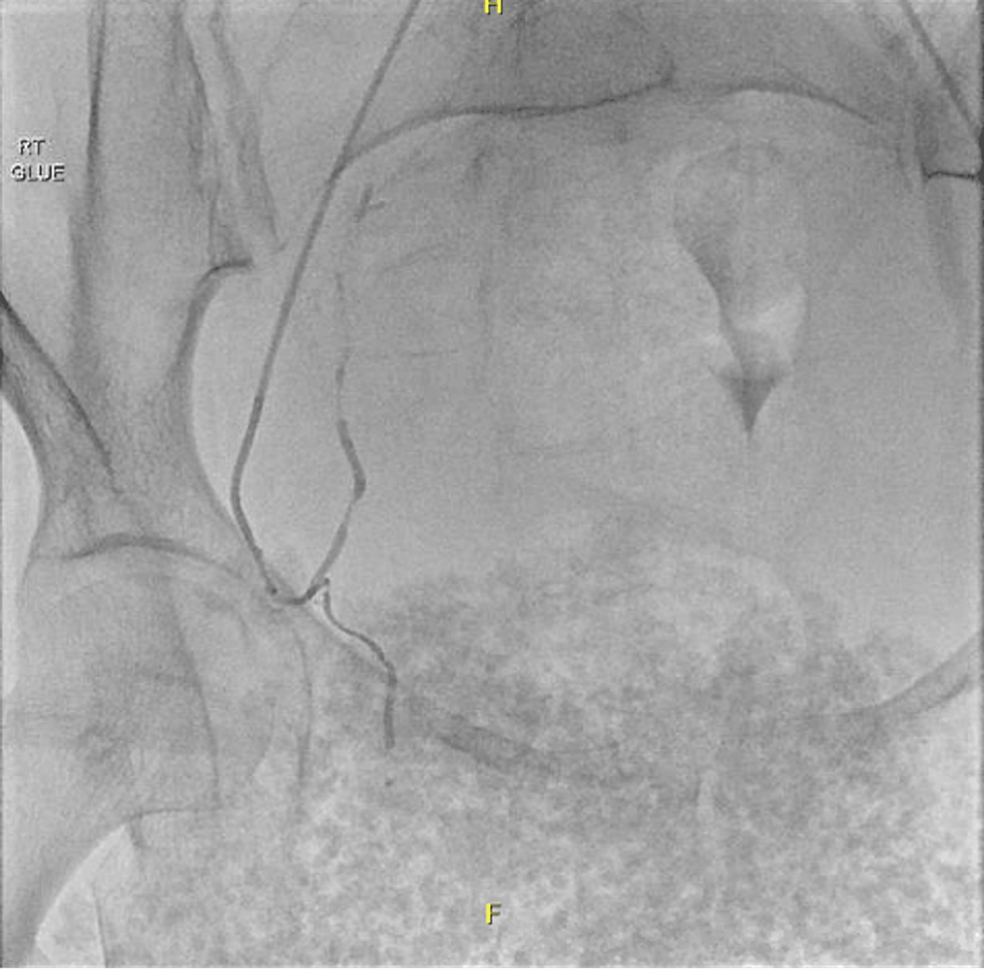
A follow-up angiography demonstrating no further bleeding after embolization of the corona mortis

After the CT imaging findings, the patient underwent a pelvic angiogram with embolization. Left femoral artery catheter insertion was conducted, advancing to the right common iliac artery, followed by angiography. The angiogram displayed an active extravasation at the right corona mortis (Figure 3), denoting an anastomosis between the IEA and OA. Subsequently, the corona mortis was selectively catheterized using a microcatheter, followed by embolization with Histoacryl glue (Figure 4). Successive follow-up angiograms of both IIA and EIA showed no appreciable further active extravasation. The patient demonstrated a good tolerance to the procedure and was thereafter transferred to the ward in a stable condition for ongoing care.

**Figure 5:**
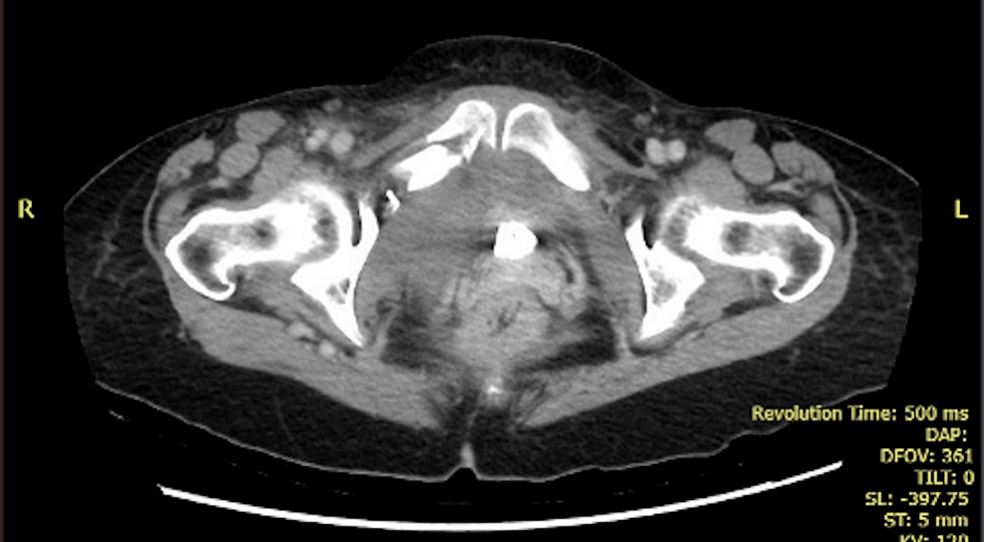
A CT of the abdomen and pelvis with contrast exhibiting an absence of delayed contrast extravasation in the pelvic hematoma CT: computed tomography

The patient was initially admitted to a high-dependency unit for the early days postprocedure, where her vital signs and blood count were continuously monitored. Following the procedure, she was vitally stable, with a hemoglobin level of 9.6 g/dL. For her pelvic fracture, a conservative approach was adopted, entailing a two-week period of bed rest with gradual mobilization as tolerated. One week after admission, a repeat CT of the abdomen and pelvis with contrast revealed an absence of delayed contrast extravasation within the pelvic hematomas (Figure 5).

Her condition gradually improved, prompting the transition to wheelchair mobilization before eventual discharge. At the time of discharge, her hemoglobin level had risen to 11.6 g/dL. The patient was discharged after three weeks with a prescription for regular painkillers, a 30-day course of low-molecular-weight heparin as prophylaxis against deep vein thrombosis, and specific instructions for physiotherapy rehabilitation. Additionally, she was scheduled for a follow-up appointment in the outpatient department in a month's time. This holistic approach ensures support for patients throughout their recovery and addresses any ongoing needs or concerns.

**Figure 6:**
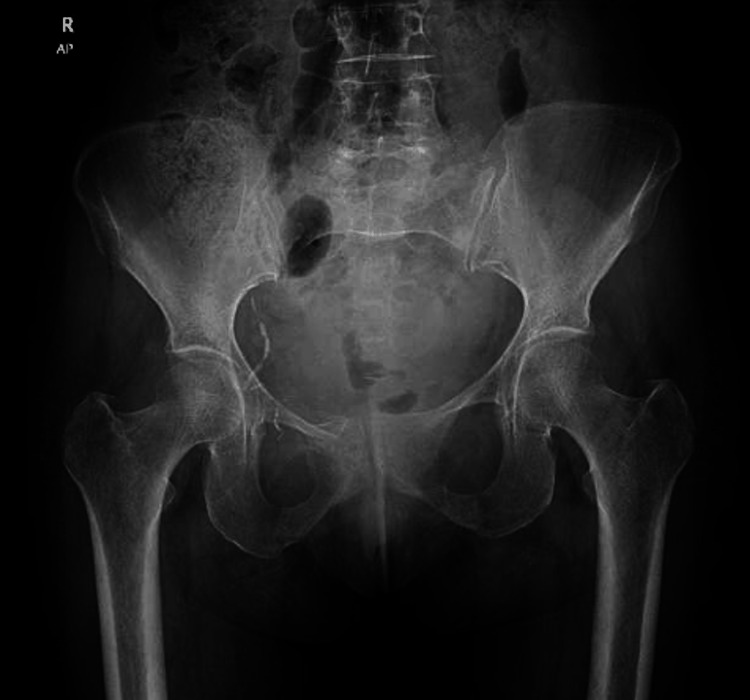
Pelvic X-ray displaying the initial fractures in the healing process with a well-healed left IPR fracture with callus formation and a misaligned right SPR. Additionally, linear density can be seen projected on the right side of the pelvis, likely postembolization IPR: inferior pubic rami; SPR: superior pubic ramus

One month later, at the initial follow-up, the patient persisted in mobilizing in a wheelchair. Repeat X-rays showed satisfactory healing, with a well-uniting left IPR fracture exhibiting callus formation, albeit accompanied by a malunited right SPR (Figure 6). A month later, during the consecutive appointment, she exhibited significant improvement, mobilizing fully with minimal pain that was well tolerated without the necessity of regular painkillers. The positive recovery observed allowed for an extended interval before the next scheduled follow-up, which was set for three months, indicating a favorable outcome in her journey.

## Discussion

Fractures of the pubic rami resulting from low-energy trauma are prevalent among the elderly, with an annual incidence of 26 per 100,000 in individuals aged 60 years and above [5]. In contrast to analogous fractures observed in younger populations, typically resulting from high-energy trauma, these fractures seldom exhibit life-threatening complications [9]. Treatment predominantly adopts a conservative approach, entailing mobilization as tolerated and the administration of analgesia. Consequently, vigilant monitoring is typically deferred in such cases. However, our case emphasizes the potential for even benign fractures to precipitate life-threatening bleeding, warranting a heightened index of suspicion.

Elderly individuals are predisposed to hemorrhage due to compromised hemostasis, contingent upon local vasospasm, tamponade, and blood clotting ability. Atherosclerosis contributes to diminished vasospasm and vessel compliance. Moreover, the elderly exhibit poor soft-tissue turgor, reducing the tamponade effect on pelvic contents. The confluence of polypharmacy and anticoagulant use further impedes blood clotting [10,11].

**Figure 7:**
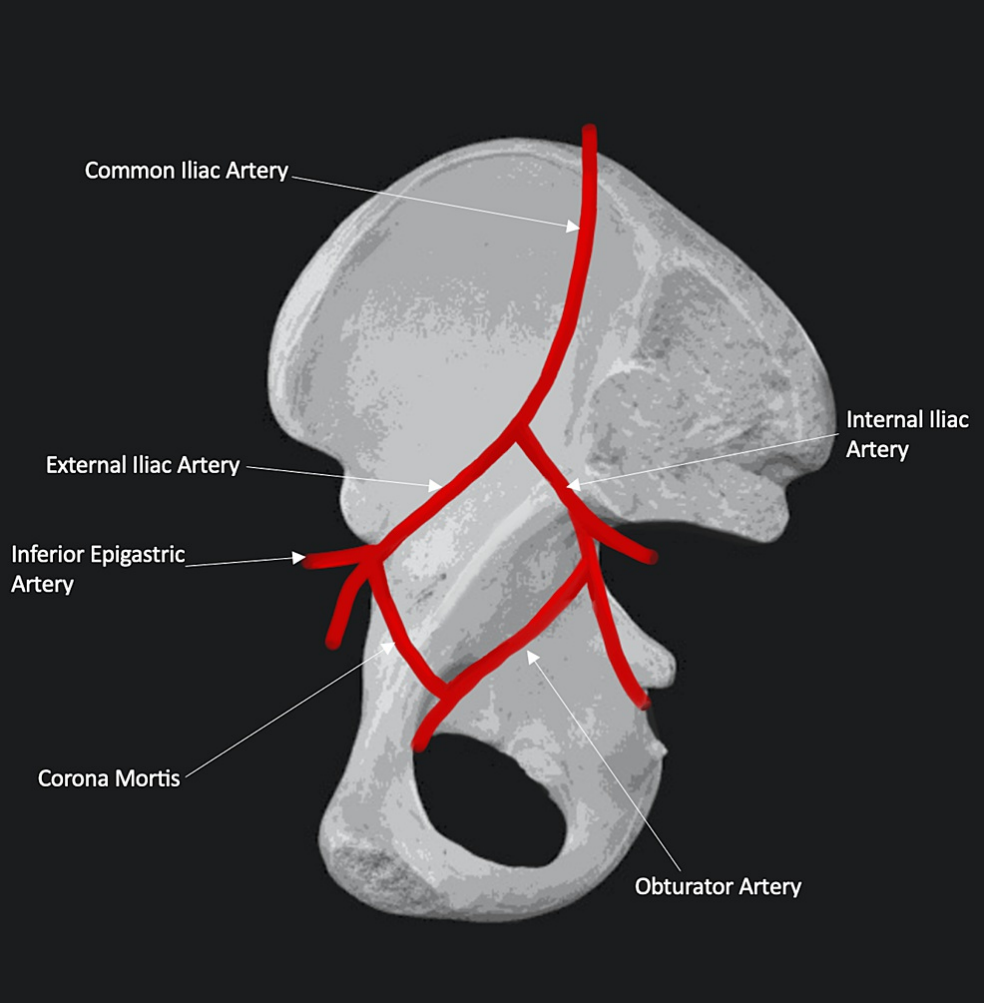
An illustration of the corona mortis as described in our patient (created in iArtbook)

Looking at the pelvic vascular anatomy, the IEA originates from the EIA proximal to the inguinal ligament. It gives off a pubic branch that descends to the internal surface of the pubis, wherein, in certain instances, it establishes an anastomosis with the OA, a branch stemming from the IIA, a phenomenon recognized as corona mortis (Figure 7) [1-3]. Corona mortis is delineated as an anastomotic variant between the OA and the EIA or IEA or vein, concurrently involving other vessels. This phenomenon is categorized by the Rusu et al. system into three principal classifications: I-arterial, II-venous, and III-combined, with a heightened prevalence of venous connections [1]. Cadaveric studies have elucidated an anastomosis between the external iliac and obturator systems in 84% of specimens, with 34% having arterial connections, 70% venous connections, and 20% exhibiting both [2]. Being close to the SPR, it is susceptible to compromise even with stable fractures, potentially culminating in catastrophic hemorrhage, justifying its name, which translates to ‘‘the crown of death’’ [3,4].

Our patient presented with early hemodynamic instability, facilitating a prompt response, while similar cases documented in the literature showcased delayed deterioration, highlighting the imperative of admitting patients for close monitoring [4,12-17]. Physical examination is pivotal in identifying critical cases with a heightened likelihood of arterial injury. Red flags such as bruising, abdominal, groin, perineal swelling, and pain must be deliberately observed [12]. A drop in hemoglobin level serves as another alerting sign, prompting early investigation of potential bleeding by serial monitoring of hemoglobin levels every four to six hours and additional assessments after 24 hours [14]. In a study, three predictors of bleeding in patients with stable fractures were identified: a hematocrit (Hct) of 30% or lower, the presence of a pelvic hematoma on CT, and a systolic blood pressure of 90 mmHg or lower [8]. Consequently, presuming that hypotension and anemia, in the absence of intra-abdominal hemorrhage, denote pelvic bleeding is justified until proven otherwise. These signs should prompt physicians to order additional imaging modalities such as CT scans or ultrasounds. It is noteworthy that the FAST scan yielded negative results in our patient. CT scan remains the modality of choice, demonstrating superior accuracy in identifying hematomas and arterial bleeds, and has been associated with reduced mortality [16,18]. The CT scan can also precisely delineate the location of hematoma and minor bony fractures and rule out life-threatening intra-abdominal injuries. In this patient, embolization of the corona mortis, an anastomosis by IEA and OA in our case, was executed using the contralateral femoral artery to mitigate potential interference of the hematoma with initial catheterization, a technique endorsed by the literature [19]. Follow-up angiography was conducted to verify the absence of further extravasation that could arise due to this anastomotic variant.

Management of hemorrhage resulting from significant major pelvic trauma can be approached through preperitoneal pelvic packing or percutaneous angiographic embolization, with the latter being the preferred method [6-8]. Packing is typically employed when angiography is unavailable or whenever emergency surgery precedes angiography [8]. In the elderly population, embolization is deemed safe and exceptionally effective, even when severe shock symptoms are present [16].

## Conclusions

In conclusion, while life-threatening bleeding is a rare complication following stable pelvic fractures, it is imperative for physicians to maintain a heightened threshold for suspicion, particularly in the elderly. These patients require admission with an emphasis on a comprehensive physical examination, and serial monitoring of hemoglobin levels. Subsequent consideration of a CT scan based on clinical findings is warranted.
